# Hybrid Parallel Compliance Allows Robots to Operate With Sensorimotor Delays and Low Control Frequencies

**DOI:** 10.3389/frobt.2021.645748

**Published:** 2021-06-16

**Authors:** Milad Shafiee Ashtiani, Alborz Aghamaleki Sarvestani, Alexander Badri-Spröwitz

**Affiliations:** Dynamic Locomotion Group, Max Planck Institute for Intelligent Systems, Stuttgart, Germany

**Keywords:** legged robots, parallel and passive compliance, hybrid actuation and leg design, sensorimotor delay, Feedback, latency, parallel elastic actuation

## Abstract

Animals locomote robustly and agile, albeit significant sensorimotor delays of their nervous system and the harsh loading conditions resulting from repeated, high-frequent impacts. The engineered sensorimotor control in legged robots is implemented with high control frequencies, often in the kilohertz range. Consequently, robot sensors and actuators can be polled within a few milliseconds. However, especially at harsh impacts with unknown touch-down timing, controllers of legged robots can become unstable, while animals are seemingly not affected. We examine this discrepancy and suggest and implement a hybrid system consisting of a parallel compliant leg joint with varying amounts of passive stiffness and a virtual leg length controller. We present systematic experiments both in computer simulation and robot hardware. Our system shows previously unseen robustness, in the presence of sensorimotor delays up to 60 ms, or control frequencies as low as 20 Hz, for a drop landing task from 1.3 leg lengths high and with a compliance ratio (fraction of physical stiffness of the sum of virtual and physical stiffness) of 0.7. In computer simulations, we report successful drop-landings from 3.8 leg lengths (1.2 m) for a 2 kg quadruped robot with 100 Hz control frequency and a sensorimotor delay of 35 ms.

## 1 Introduction

Animals use muscle-tendon networks, which they control by spinal circuits, the brainstem, and with sensory feedback to produce joint torque and work for legged locomotion (Forssberg et al., 1977; Grillner and Wallen, 1985; Biewener, 1989; Ijspeert, 2008; Takakusaki et al., 2016; Stratmann et al., 2018). The response time for muscle action caused by an external stimulus is related to axonal conduction velocity and animal body weight, and the resulting sensorimotor delay can be as slow as 41 ms in a 4 kg, cat-sized animal (More et al., 2010; Franklin and Wolpert, 2011; More and Donelan, 2018). House cats run with up to 5 Hz locomotion frequency (Bertram et al., 2014). At an assumed duty cycle of 0.4 the stance phase lasts 80 ms, and the animal would be sensor-blind for half its stance phase, i.e., during the entire force ramp-up time. We often assume feedback to be critical in challenging conditions like in rough terrain locomotion. However, running birds and other animals traverse hidden perturbations with ease, albeit limited sensorimotor capabilities (Daley et al., 2006; Ernst et al., 2018).

**FIGURE 1:**
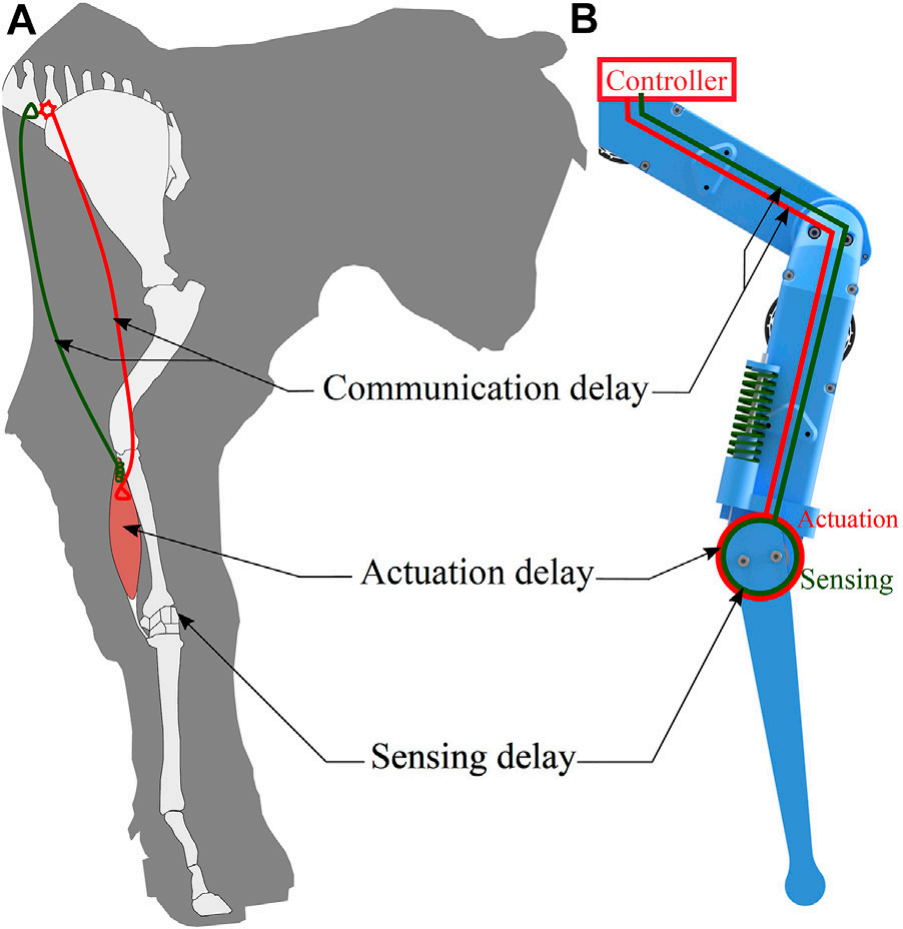
**(A)** Animal locomotion control is subject to sensorimotor delays from sensing, communication, and actuation. The drawing is loosely inspired by Figure 1 of More and Donelan (2018). **(B)** Robots typically have lower intrinsic delays from electronic sensing and communication. Instead, delays are caused, for example, by filtering noisy data. We systematically tested robot controllers with varying sensorimotor delay and control frequency. We hypothesized that a hybrid system comprised of passive joint elasticity and parallel active joint stiffness can reject sensorimotor delays robustly, for appropriate compliance ratios.

Animal locomotion control is simplified by a morphology with tendons and muscles with intrinsic physical stiffness (Alexander, 1990; Blickhan et al., 2007a). Physical elasticities mounted serially like tendons can lead to under-actuation and reduced controllability. However, animals show no obvious signs of decline in robustness, responsiveness, or agility. Many muscle-tendons are part of more extensive networks with parallel muscle-tendon units, requiring sensorimotor coordination (Lombard, 1903; Hutchinson et al., 2005). This raises two questions: For parallel mounted active and passive stiffness, how do animals deal with significant sensorimotor delays (Figure 1A)? And how are legged robots impacted (Figure 1B)? This section briefly reviews concepts from biomechanics and legged robotics dealing with sensorimotor delays, the control of leg forces, especially at leg impacts, and active and passive joint stiffness. In the main part of this work we present a robotic proof-of-concept characterizing parallel active and passive stiffness as one source of robustness against adverse conditions for feedback controllers.

Inspired by animal morphology and passive and active leg stiffness, legged robot designs often include mechanical springs (Nasiri et al., 2016; Ambrose and Ames, 2020). Series elastic actuation (SEA) can simplify control, improve robustness and interaction safety, and protect actuators from overloads (Raibert et al., 1984; Robinson et al., 1999; Pratt and Krupp, 2004; Hutter et al., 2011; Calanca et al., 2015; Hutter et al., 2016; AhmadSharbafi et al., 2020). Designs with parallel mounted springs and actuators (parallel elastic actuation, PEA) can increase leg forces, improve locomotion energy efficiency, and reduce actuator loading (Gunther et al., 2015; Niehues et al., 2015; Plooij et al., 2016; Yesilevskiy et al., 2016; Liu et al., 2018; Toxiri et al., 2018; Yesilevskiy et al., 2018; Roozing et al., 2019; Ambrose and Ames, 2020). Combined parallel and serial elastic designs have been proposed, leading to reduced peak torques and improved locomotion applicability (Grimmer et al., 2012). Leg stiffness is altered mechanically in several ways; decoupling actuator and spring action during the locomotion cycle can simplify control and improve energy efficiency (Wiggin et al., 2011; Spröwitz et al., 2013). Variable elastic mechanisms augment physical stiffness for efficient actuation (Choi et al., 2011; Mathijssen et al., 2014; Braun et al., 2016). Until today, it remains challenging to effectively alter and rapidly manipulate compliance under high loads while keeping the mechanisms compact, robust, and lightweight.

Serial and parallel elastic-legged robots can locomote by feed-forward control and without system state knowledge from feedback (Iida and Pfeifer, 2004; Narioka et al., 2012; Spröwitz et al., 2018; Ruppert and Spröwitz, 2019). However, passive, compliant designs are under-actuated and show limited controllability. Parallel elastic designs can maintain good control authority; when controllability is more needed than spring-based natural dynamics, the actuator overrides the spring’s action (Verstraten et al., 2016). Usually, parallel elastic legs are designed with strong springs providing all essential torques and forces. Consequently, strong, relatively heavy, and fast actuators are required to override springs.

Legged robots with proprioceptive actuation and sensing and quasi-direct drives feature the highest control authority, compared to passive and partially actuated designs (Seok et al., 2012; Ding and Park, 2017; Park et al., 2017). These legged machines are agile and fast, they jump high, and land robustly (Park et al., 2017; Grimminger et al., 2020). From a sensorimotor perspective, proprioceptive actuators require 1) low communication and control delays in the range of a few milliseconds allowing 2) high-frequency control above 500 Hz, 3) accurate force and joint speed sensing, 4) and precise touch-down sensing (Bledt et al., 2018; Grimminger et al., 2020; Li et al., 2020). Not all conditions are always met, especially in unknown terrain and during harsh touch-downs, when actuator gains are changed, and when sensor noise indirectly causes feedback delays (Hubicki et al., 2016; Hammoud et al., 2020).

Robot force sensors are affected by leg impacts loading legs from zero to multiple body weights in a few ten milliseconds, and leading to wobbling masses (Günther et al., 2003; Mo et al., 2020). Impact vibrations transfer to the sensor’s mechanics and appear as sensor noise requiring processing (Spröwitz et al., 2018; Grimminger et al., 2020). Low-noise leg force sensors are being developed, yet there remains a trade-off between sensitivity and specificity, sensor noise from impacts, and sensor weight and complexity (Ananthanarayanan et al., 2012; Hutter et al., 2014; Grimminger et al., 2020; Ruppert and Badri-Spröwitz, 2020). Noisy force data can be filtered to identify touch-down and leg loading uniquely, but filtering adds to the overall sensorimotor delay; for example, delays of 31 ms are documented to uniquely identify touch down with proprioceptive sensing (Grimminger et al., 2020). Monitoring the deflection of physical joint elasticity provides alternative leg loading information, for example, for virtual model control (Pratt et al., 1997). Virtual damping assumes precise speed estimation, but numerically differentiating noisy signals requires filtering for sufficiently smooth signals, leading to feedback delay (Flacco et al., 2012; Hammoud et al., 2020).

Robot-internal electrical communication is limited only by the speed of light, and with relatively short wire lengths communication delays are minimal. Contrary, teleoperation between operator and legged robot over long-distance can lead to significant feedback delays (Varkonyi et al., 2014). Dedicated force feedback control can robustly deal with limited delays; beyond that, control destabilizes (Lee and Spong, 2006; Shafiee-Ashtiani et al., 2017; Shafiee et al., 2019). The applicability of high-level locomotion planners is related to control frequency and therefore also to sensorimotor delay; current planners run on time for control frequencies above 100 Hz (Ponton et al., 2018; Mastalli et al., 2020). Legged robots intrinsically tolerating low control frequencies are therefore good candidates for complex online locomotion planners.

Besides virtual and physical springs, both virtual and physical damping have been applied to control legged locomotion, also as part of impedance control (Seok et al., 2012; Boaventura et al., 2013; Nagayama et al., 2016; Park et al., 2017; Heim et al., 2020; Mo et al., 2020). By dissipating excess potential and kinetic energy and producing damping forces, leg reaction forces are adapted, and post-impact oscillations are reduced (Blickhan et al., 2007a; Haeufle et al., 2014; Semini et al., 2015). Virtual damping control requires precise speed estimation, which makes the method brittle in the presence of sensor noise (Bledt et al., 2018; Hammoud et al., 2020). Mechanical leg dampers are immune to feedback delays and sensor noise but must actively be switched off when not required (Mo et al., 2020).

In animals, upper limb control is subject to sensorimotor delays, like during manipulation tasks. Humans and other animals manipulate objects by exploiting muscle-tendon elasticities, effectively changing joint stiffness (Franklin et al., 2004). Antagonistic pairs of muscle-tendons can be prestressed by feed-forward (‘preflex’) control, leading to increased joint stiffness for a given posture independent from feedback delay, but with limited movement range (Hogan, 1984; Crevecoeur and Scott, 2014). Alternatively, reflexes can alter joint stiffness. Mouel and Brette (2019) show that increased joint stiffness should be compensated for by reduced sensorimotor gains; otherwise, delayed feedback leads to unstable behavior. Setting joint impedance through feed-forward sensorimotor commands might allow stable upper limb postures with noisy state estimation (Berret and Jean, 2020). Upper limb manipulation and lower limb locomotion tasks differ in their respective loading scenarios. Most manipulation tasks are continuous, while legged locomotion is always hybrid and non-continuous. Leg forces and loading times depend on body weight and drop height. The leg forces in this work ramp up from zero to body weights within 0.1 s and lead to joint angle changes above 45°. End-effector forces during manipulation are typically within the range of the object’s weight instead of the user’s body weight (Crevecoeur and Scott, 2014).

In this work, we aim to merge two diametrical principles while maintaining their best properties; 1) Passive leg joint compliance that works without feedback and at low control frequency, and 2) active joint compliance providing control authority. We hypothesize that, for a given robot design and locomotion task, there exists a range of compliance ratios—a ‘hybrid’ range—that works best despite significant feedback delays and low control frequencies.

This work uniquely contributes as follows; We systematically characterized the full range of active-to-passive parallel compliance ratios for a given total leg joint compliance. We simulate adverse controller conditions in simulated and hardware drop landings, including significant feedback delays, low control frequencies, and varying duty cycles. Previous work in parallel-elastic legged robotics typically investigated parallel compliance with high-frequency and low delay actuation (Mazumdar et al., 2016).

In Section 2, we present a stability analysis of a simplified model in the presence of sensorimotor delays, for two ratios of parallel compliance. We then present computer simulations and hardware experiments and investigate the effect of control frequencies, sensorimotor delays, and duty cycles on a robot leg with varying ratios of parallel compliance, for drop-landings (Section 3). We also characterize a simulated quadruped robot made of four of these legs, for multiple drop-landing heights. We discuss the work in Section 4, and conclude in Section 5.

## 2 Materials and Methods

We quantify the total (sum of) system compliance as active compliance in parallel to passive (spring-based) compliance, acting at the knee joint (Figure 1B):

$$K_{\text{total}}=K_{\text{active}}+K_{\text{passive}}$$ (1)

where $K_{\text{passive}}$ [Nm/rad] is the joint’s passive rotational stiffness, $K_{\text{active}}$ [Nm/rad] is the joint’s active, virtual, rotational stiffness produced by the actuator. $K_{\text{total}}$ [Nm/rad] is the summed up rotational joint stiffness. We define a ‘compliance ratio’ $\lambda_{\text{passive}}$ as the ratio of passive stiffness and total stiffness:

$$\lambda_{\text{passive}}=K_{\text{passive}}/K_{\text{total}}$$ (2)

Hence, for a compliance ratio $\lambda_{\text{passive}}=0.1$ the knee spring supplies 10% of the knee torque to carry the robot, and the knee actuator supplies the remaining 90%. A $\lambda_{\text{passive}}$ of 1.0 indicates a knee joint with a physical spring and no motor.

### 2.1 Theoretical Analysis of a Simplified, Reduced Model of an Actuated Pendulum

We analyzed a simplified system with parallel compliance, to analytically quantify the effects of sensorimotor delays. The reduced order model consists of a strut-like leg mounted as a single degree-of-freedom pendulum and represents a simplified robot lower leg (Figure 4A). The equations governing the pendulum motion are:

$$I\ddot{\theta }\;\;+\;mgL\cdot sin\left(\theta -\theta_{0}\right)+K_{\text{passive}}\left(\theta -\theta_{0}\right)+D\dot{\theta }=\tau_{\text{m}}$$ (3)

where D=0.14 Nms/rad is the system damping, $K_{\text{passive}}$ is the stiffness of the parallel compliant element, L=0.16 m is the center of mass distance to the pivot point, m=0.5 kg is the mass, $I=mL^{2}$ is the moment of inertia, *g* is the standard gravity, and $\theta_{0}$ is the equilibrium joint angle of the relaxed spring. We set a total stiffness of $K_{\text{total}}=1.15\;\text{Nm}/\text{rad}$. The instantaneous joint angle is *θ*, and $\tau_{\text{knee}}$ is the knee joint control torque input, implemented as active compliance:

$$I\ddot{\theta }\;\;+mgL\cdot sin\left(\theta -\theta_{0}\right)+K_{\text{passive}}\left(\theta -\theta_{0}\right)+D\dot{\theta }=-K_{\text{active}}\left(\theta_{\text{feedback}}-\theta_{0}\right)$$ (4)

where $K_{\text{active}}$ is the active motor compliance. The sensor reads the joint angle $\theta_{\text{feedback}}$. We assume a small enough angular deviation of the pendulum around the equilibrium point: $\sin\left(\theta -\theta_{0}\right)≃\left(\theta -\theta_{0}\right)$, which allows to write Eq. 4 as a linear differential equation. We converted Equation 4 to the Laplace domain and incorporated a fixed feedback time delay $t_{\text{d}}$ of the control input (active compliance). The resulting closed-loop system transfer function can be presented in the frequency domain as:

$$\frac{{\text{Θ}}_{\text{s}}}{{\text{Θ}}_{\text{ds}}}=\frac{K_{\text{active}}e^{-t_{d}s}+mgL+K_{\text{passive}}}{s^{2}I+Ds+K_{\text{active}}e^{-t_{d}s}+K_{\text{passive}}+mgL}$$ (5)

We linearized the system’s exponential time delay term with a third-order Padé approximation. A system pole analysis of this simple system provides an intuitive understanding of the effects of two compliance ratios for a given total joint stiffness on closed-loop stability, and for given sensorimotor delays.

### 2.2 Computer Simulation of Articulated Robot Legs

We characterized a single, articulated robot leg with hybrid joint compliance. Drop landings are one of the most challenging tasks due to high, impulse-like ground reaction forces, and nonlinear and hybrid leg loading. Drop landing is similar to a step response perturbation, which is a conventional control theory tool to characterize black box systems. We computer simulated the robot leg in PyBullet (Coumans and Bai, 2019), and performed extensive drop-landing simulations for a broad range of sensorimotor delays, duty cycle frequencies, and $\lambda_{\text{passive}}$. We simulated a single leg and a quadruped robot, both modified from the open-source quadruped robot Solo (Grimminger et al., 2020).

**FIGURE 2:**
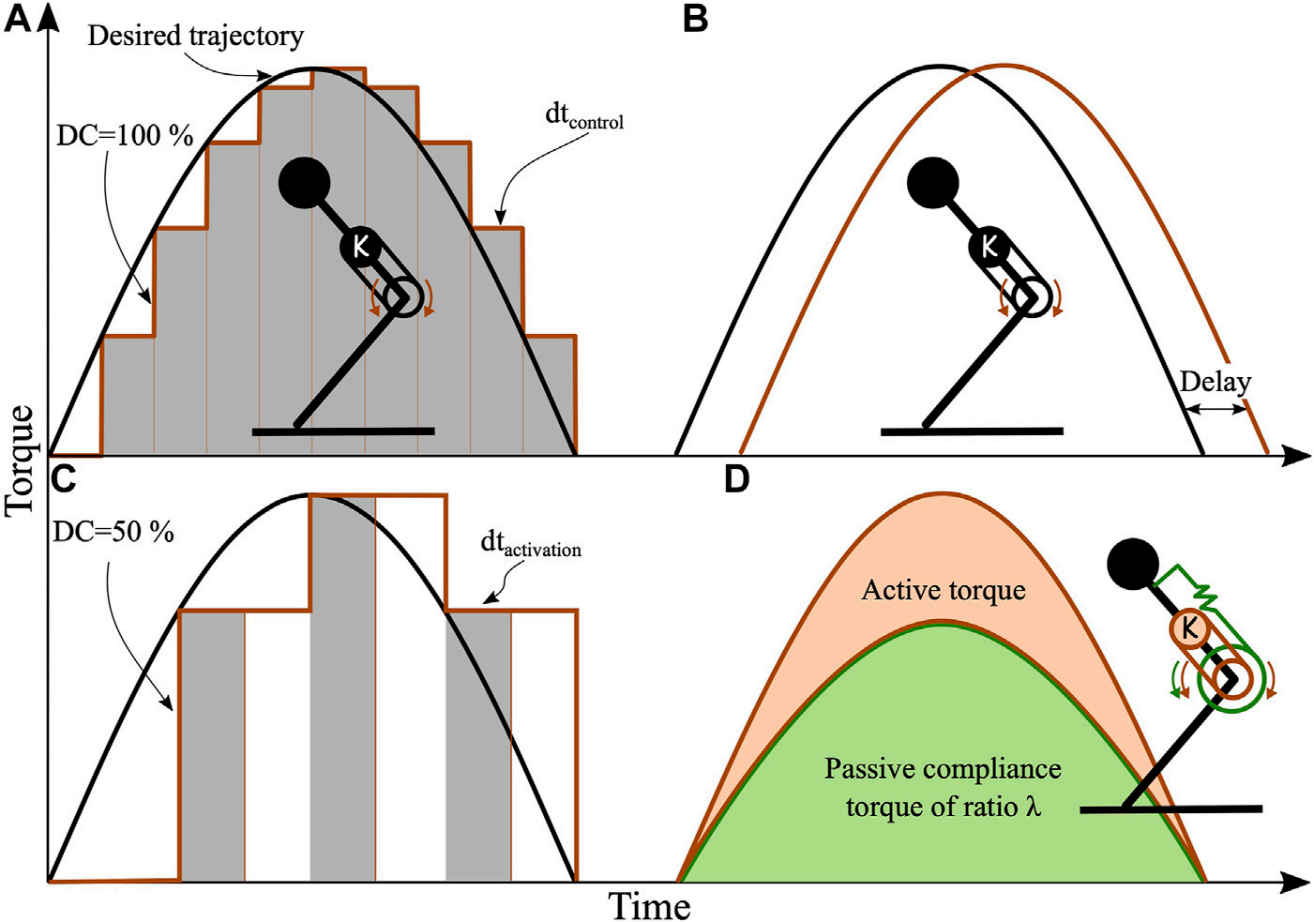
Knee motor command for different combinations of control frequency, duty cycle, and sensorimotor delay. **(A)** A 100% duty cycle at low control frequency. **(B)** A set sensorimotor delay between the desired knee output torque, and the commanded output torque. **(C)** A 50% duty cycle. **(D)** An example for a compliance ratio of $\lambda_{\text{passive}}\approx 0.75$ is shown. The mechanical knee spring produces three quarter of the total knee torque (green). The knee actuator is programmed as a virtual spring producing the remaining torque (brown).

In Figure 2, we show the control and sensorimotor strategies tested. The black curve is the schematic, desired knee motor torque trajectory. The control frequency (step-like, brown line) is measured in commands per second. For reference, the control frequency of proprioceptive actuation in legged robots is often around 1 kHz, i.e., a cycle period takes $dt_{\text{control}}=\frac{1}{f}=1\;\text{ms}$. We are especially interested in investigating scenarios with control frequencies well below 1 kHz.

Torque is applied with three strategies; First, the activation duration $dt_{\text{activation}}$ is defined as the time period between control commands, i.e., $dt_{\text{activation}}=DC\;\;\times \;\;dt_{\text{control}}$. The activation duration lasts at least 1 ms and at most $\frac{1}{f}$. For $dt_{\text{activation},\text{min}}$, the control command is applied for a period of 1 ms and then reset to zero. For $dt_{\text{activation},\text{max}}$, the actuator will maintain its value until the control command is updated (Figure 2A, brown line). Second, we applied a sensorimotor delay to the control command (Figure 2B). Third, the force-activity relationship of muscles is not fully understood (Roberts and Gabaldón, 2008), and we included tests with varying duty cycles, defined as the fraction of $dt_{\text{control}}$ with a non-zero actuator torque (Figure 2C).

The active compliance controller knee joint input is:

$$\tau_{\text{knee},\text{motor}}=K_{\text{total}}\left(1-\lambda_{\text{passive}}\right)\left(\theta_{\text{feedback},\text{knee}}-\theta_{0,\text{knee}}\right)$$ (6)

To simulate the spring in PyBullet, we implemented a knee joint spring torque:

$$\tau_{\text{knee},\text{spring}}=K_{\text{total}}\left(\lambda_{\text{passive}}\right)\left(\theta_{\text{knee}}-\theta_{0,\text{knee}}\right)$$ (7)

### 2.3 Setup Hardware Experiments

**FIGURE 3:**
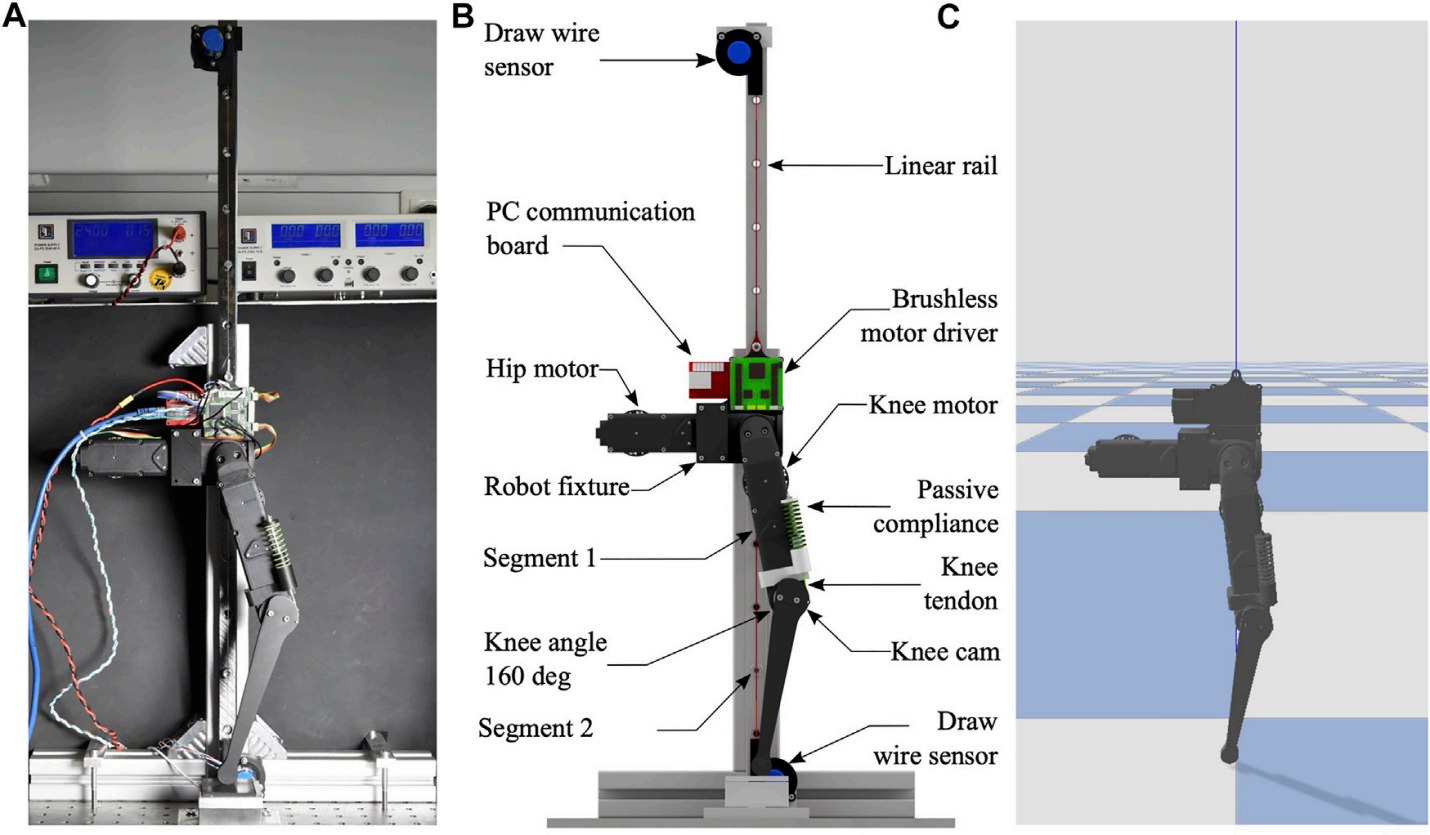
Experimental setup. **(A)** The 2-DOF hybrid compliant leg. The one-directional spring (passive compliance) extends the knee joint via a knee tendon and a knee pulley. Knee springs with varying stiffness were mounted during the experiments, supporting between 0 and 100% robot’s weight. A rail guides the robot’s vertical drop, and a pair of potentiometers measures the robot’s height. The knee motor produces torques in parallel to mounted knee spring. **(B)** Setup details, computer aided drawing. **(C)** The Unified Robot Description Format (URDF)-based model of the hybrid compliant robot leg, simulated in PyBullet.

We modified a single leg of the eight degree-of-freedom (8-DOF), open-source, quadruped robot ‘Solo’ (Grimminger et al., 2020). The leg has two active degrees of freedom, one at the hip and one at the knee. Both leg segments are 0.16 m long, the lower leg mounts a semi-circular foot of 15 mm radius. A brushless motor (Antigravity MN4004-kv380, T-Motor) drives a two-stage belt transmission with an overall 9:1 gear ratio for each active joint. An optical encoder (AEDT-9810-T00, Avago) measures the motor’s rotor position, which is recalculated into joint angles. We mounted physical springs in parallel to the knee joint (SWY 16.5–30 for $\lambda_{\text{passive}}=1.0$, SWY 16.5–45 for $\lambda_{\text{passive}}=0.67$, SWY 16.5–80 for $\lambda_{\text{passive}}=0.37,$ Misumi). The spring’s tendon inserts into a knee joint pulley with radius 18.9 mm (Figure 3B). The spring mount allows rapid exchange of springs between experiments.

To simplify the touch-down scenario, the robot leg was dropped guided by a vertical rail (Figure 3A). The hip joint was constrained to follow half of the knee joint angle at all times, controlled by a position controller creating foot contact vertically below the hip joint. We recorded the vertical hip position with two draw-wire sensors (LX-PA-40, WayCon) mounted above and below the robot, to cancel out single sensor force bias. The hip position allows quantifying the robot’s landing behavior and characterizing hybrid compliance. The hip position was sampled by an analog-to-digital (A/D) converter on the brushless motor driver board. The motor board sends motor position and vertical position data to the PC communication board, via a serial peripheral interface (SPI). The PC communication board connects the motor driver board via EtherCAT to a PC (Intel Xeon(R) W-2145 CPU, 3.7 GHz, 16 cores, 64 bit, 62.5 GB Ram, Ubuntu 18.04). We wrote a Python wrapper to control the robot. The Python wrapper timestamps and saves joint angles, motor currents, and hip height into a text file. We analyzed and plotted data in Matlab.

## 3 Result

This section initially presents results from the pendulum task. We then show computer simulation results with a single robot leg and hybrid joint compliance. We simulated quadruped-robot drops from multiple heights, and we present hardware experiment results with a single leg mounted to a vertical slider.

### 3.1 Hanging Pendulum Analysis, Simplified Model

**FIGURE 4:**
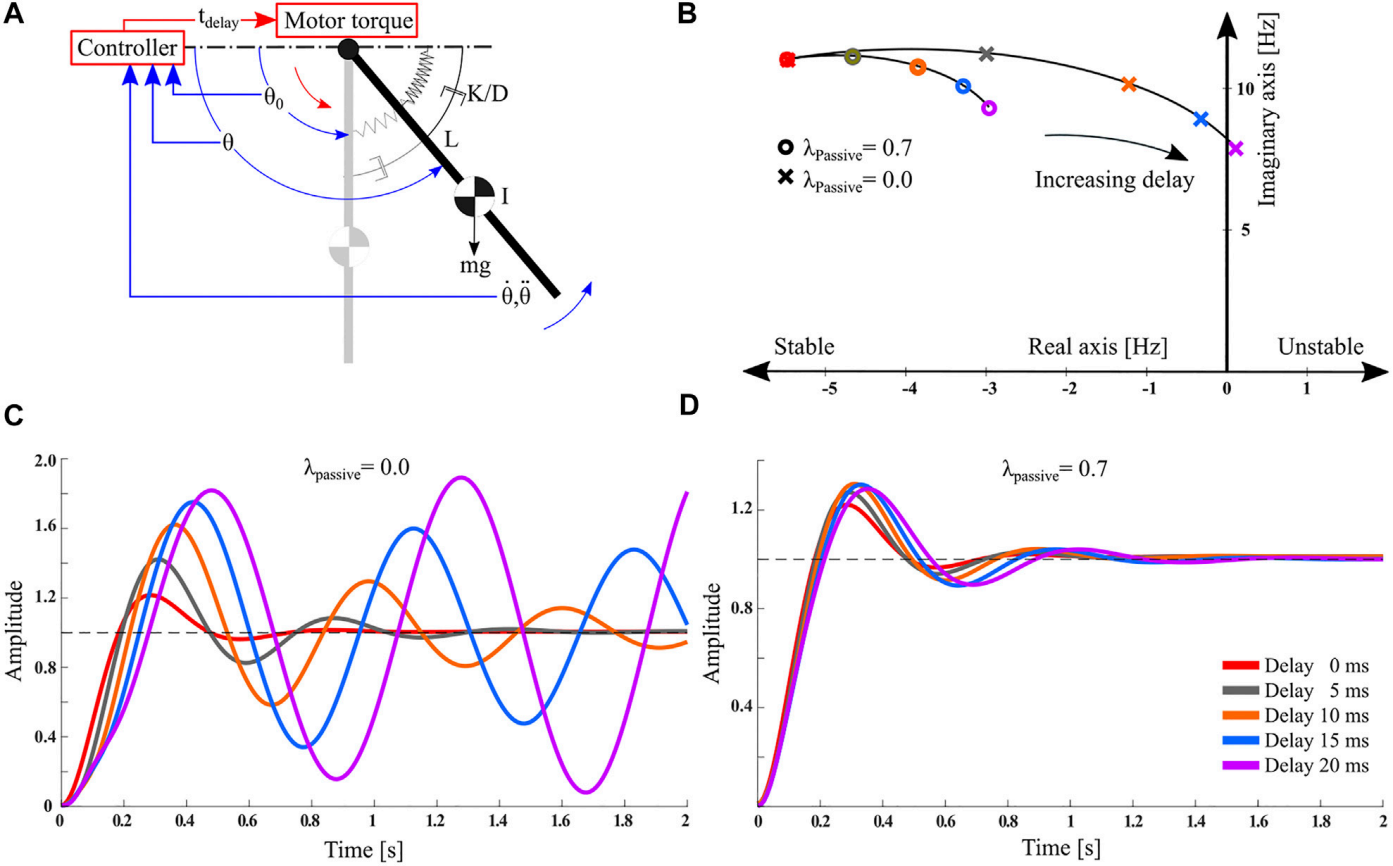
Simulation results of a simplified, single-link pendulum mounted to a parallel motor-spring combination **(A)**. Parameters are provided in Section 2.1. **(B)** Graphical pole analysis of the actuated pendulum. The effects of varying delay and compliance ratios on the system stability are shown. **(C)** The system’s step response for varying delays with $\lambda_{\text{passive}}=0.0$, and **(D)** $\lambda_{\text{passive}}=0.7$. The hybrid parallel compliance controller ($\lambda_{\text{passive}}=0.7$) is stable for all tested delays, and performs better compared to fully active actuation ($\lambda_{\text{passive}}=0.0$).

The pendulum pole analysis shows that for $\lambda_{\text{passive}}=0$ and with increasing feedback delay, the dominant system poles move from their stable region toward the unstable region at the imaginary axis (Figure 4B). For medium compliance ratios, the rate of divergence is lower. The step response indicates that increasing the sensorimotor delay with active control ($\lambda_{\text{passive}}=0.0$) leads to continuous oscillations, and resonance eventually destabilizes the system (Figure 4C). For hybrid passive compliance and a feedback delay of 20 ms, the closed-loop response is stable and smooth (Figure 4D).

The pendulum example is a simplification allowing a pole analysis with few parameters, but with an intuitive interpretation; Figure 4B shows when parameters lead to destabilization, with a clear cross-over into the unstable regime. The robot leg computer simulations in the following sections require more elaborate interpretation, but are more precise in terms of mechanics, and less simplified. Instead of continuous time analyses, time-discrete analyses are also applied for simplified systems, and we briefly provide results of a time-discrete analysis of the pendulum example in the Supplementary Material section for the interested reader.

### 3.2 Single-Leg Computer Simulation

**FIGURE 5:**
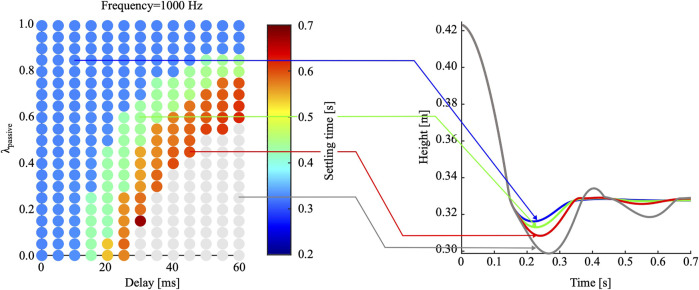
Computer simulation results: 273 drops were simulated, for the robot leg controlled with a control frequency of 1,000 Hz and a duty cycle of 100%. The compliance ratio $\lambda_{\text{passive}}$ was varied between 0 and 1 in steps of 0.05, and the sensorimotor delay between 0 and 60 ms in steps of 5 ms. The grey data points and the grey hip height trajectory show failed landings with too large settling times. All colored data points and trajectories show successful landings. Successful landings are visible for sensorimotor delays up to 60 ms, in combination with compliance ratios of $\lambda_{\text{passive}}=0.6$ and above.

We studied the effects of varying combinations of sensorimotor delay, control frequency, and compliance ratio $\lambda_{\text{passive}}$ on controller performance during landing. We initially recorded a reference hip height trajectory dropping the robot leg with $\lambda_{\text{passive}}=1.0$, which settled after 0.35 s at a hip height of 33 cm (Figure 5).

We then performed computer simulations to quantify the viability of the landing task, varying $\lambda_{\text{passive}}$ from 0.0 to 1.0 in steps of 0.05, the sensorimotor delay from 0 to 60 ms in steps of 5 ms, and sensorimotor control frequencies of 20, 50, 100, 250, and 1,000 Hz. We tested duty cycles of 25, 50, and 100%.

In PyBullet, we set joint damping values of 0.01 Nms/rad and 0.05 Nms/rad for hip and knee, respectively. A single leg weighs 0.6 kg, and the quadruped robot 2.0 kg. We chose the total knee joint stiffness so that leg length changed by 10% during the first mid-stance, after dropping it from 42.5 cm. We implemented a $\lambda_{\text{passive}}=1.0$ with a spring of stiffness K=4680 N/m acting on the knee pulley of radius r=18.9 mm, leading to a rotational stiffness of $Kr^{2}=\;\;1.67\;\text{Nm}/\text{rad}$. We defined settling time as the difference between the initiated drop time and the hip position stabilizing within a ±1% margin of the settling hip height after 3 s simulation time. We applied the Matlab function *stepinfo* for this analysis. We used twice the $\lambda_{\text{passive}}=\;\;1.0$ value as the global settling time (0.7 s) and defined 90% of the passive compliant $\lambda_{\text{passive}}=\;\;1.0$ settling hip height as minimum final hip height (30 cm).

In Figure 5, the results of 273 drop-landing simulations are shown, with varying sensorimotor delays and $\lambda_{\text{passive}}$ settings, a 100% duty cycle, and a control frequency of 1 kHz. Grey data points represent failed landings with a settling time higher than 0.7 s or too low settling hip heights. For full active actuation ($\lambda_{\text{passive}}=0.0$), and when increasing the sensorimotor delay above 25 ms, all landings fail. For $\lambda_{\text{passive}}>0.4$, the leg lands successfully in the presence of 40 ms delays. Results show that the hybrid compliant leg has successful intermediate regimes allowing for relatively large sensorimotor delays, with an appropriate combination of passive and active compliance.

**FIGURE 6:**
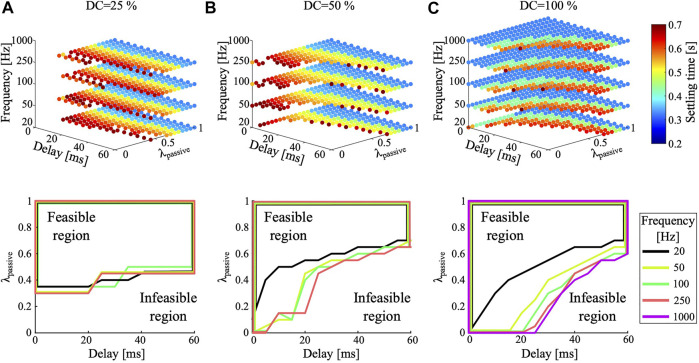
Simulation results: Dropping the hybrid actuated robot leg from a height of 42.5 cm. Parameters varied are duty cycle (DC), control frequency, system delay, and compliance ratio ($\lambda_{\text{passive}}$). The reference landing performance is the top left data point in each plot. It presents the behavior of the fully passive leg ($\lambda_{\text{passive}}=1.0$). Plots for DC = 25% and DC = 50% show no data for 1,000 Hz; with a step time of 1 ms partial duty cycles are not possible.

**FIGURE 7:**
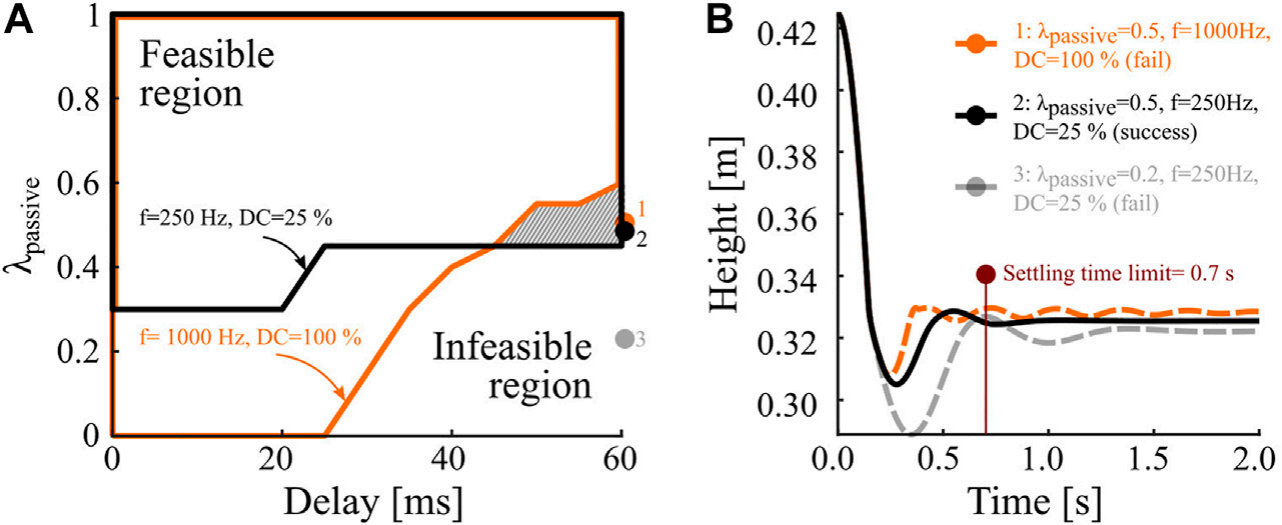
For simulations with a 25% duty cycle and 60 ms feedback delay feasible solutions are visible for compliance ratios of λ=0.5 **(B**, solid black). In the indicated overlapping parameter area **(A)**, the 100% duty cycle simulation fails with insufficient settling time **(B**, dashed orange).

We then investigated the effect of varying control frequency (20, 50, 100, 250, and 1,000 Hz) and duty cycle (25, 50, and 100%, Figure 6). Most visible is a decreasing feasible area for all three duty cycles at reduced control frequencies. Comparing duty cycles of 25 and 100% (Figures 6A,C) shows that the feasible area did change with reduced duty cycles. Low compliance ratios ($\lambda_{\text{passive}}\approx 0.2$) lead to successful landings combined with a duty cycle of 50% or the highest control frequency (1 kHz). Figure 6C shows that duty cycles of 100% at control frequencies of 100, 250, and 1,000 Hz have a similar-sized feasible region. When switching to a low control frequency (20 Hz, black line) the feasible area reduces much. For a 50% duty cycle, the feasible area changes slightly when switching between 50 and 250 Hz control frequency (Figure 6B). The biggest changes are visible when changing from 1,000 Hz to 250 Hz, and from 50 to 20 Hz. Typically, higher duty cycle values led to better results, for otherwise identical parameters. An exception is found when comparing duty cycles of 25 and 100%. The hatched area in Figure 7A indicates successful landings at low duty cycles, where high duty cycle landings failed because of hip height oscillations beyond the settling time limit (Figure 7B). For most compliance ratios above 0.6, we observe successful landings, including critical combinations of 60 ms delay and 20 Hz control frequency. All results indicate successful landing for compliance ratios equal and higher than 0.7.

### 3.3 Quadruped Computer Simulation

**FIGURE 8:**
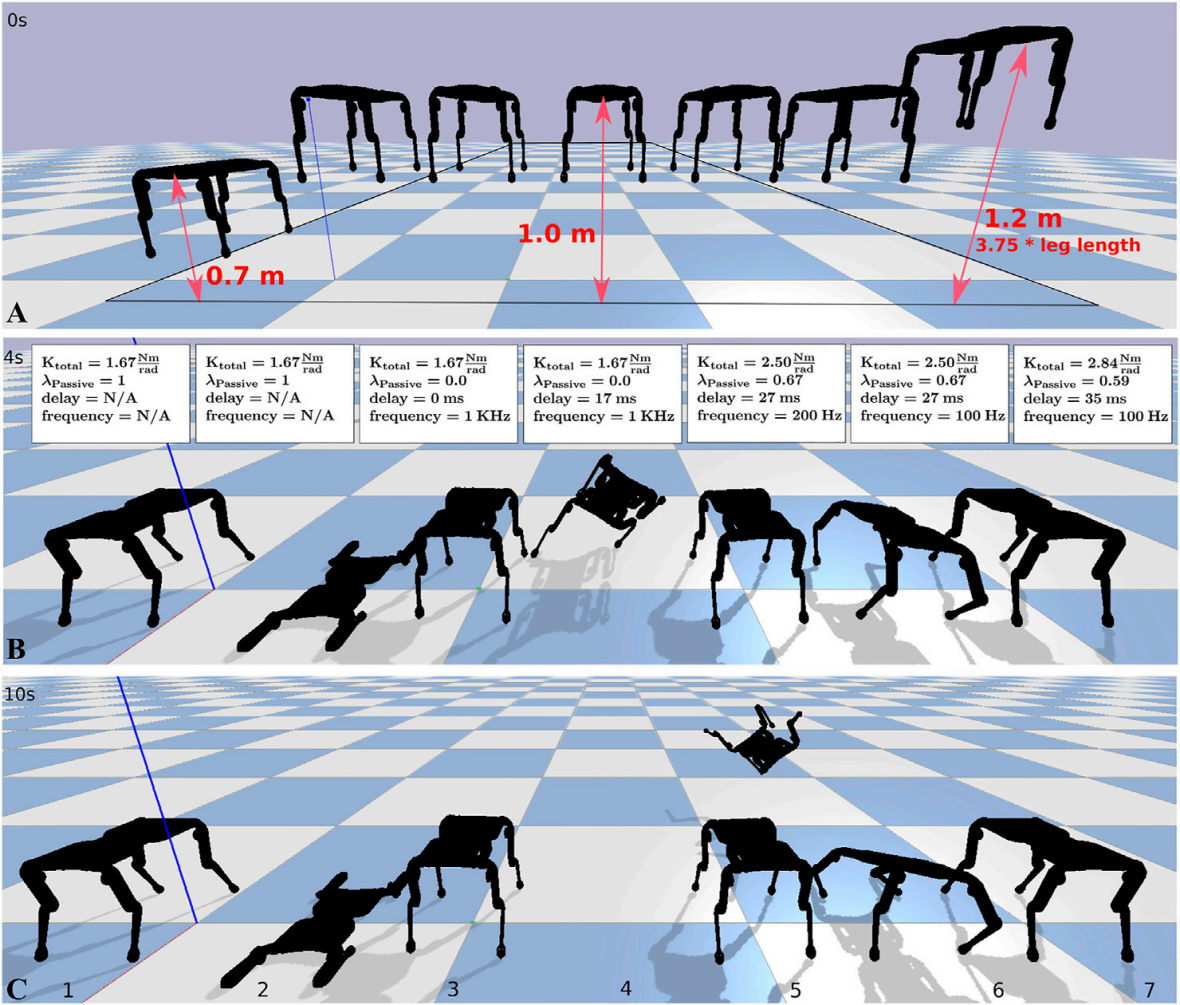
Computer simulated quadruped robots landing, in seven different scenarios, controlled with a duty cycle of 100%. **(A)** The robot’s initial drop heights are indicated with red arrows. **(B)** An intermediate robot state at 4 s simulation time. The panels also provide controller parameters. **(C)** Converged robot state after 10 s. Cases 1, 3, 5, and 7 landed successfully.

The previous single leg simulation results indicate that with high compliance ratio, robot performance becomes largely independent of sensorimotor delay, and control frequency. But fully passive compliance reduces control authority. In seven drop-landing scenarios, we altered drop height and passive and active stiffness of a quadruped robot, to characterize system and controller performance, but also to emphasize the importance of control authority (Figure 8). The duty cycle was set to 100% in all quadruped robot simulations. The simulation parameters are provided in Table 1.

**TABLE 1:** Simulation parameters of the quadrupedal robot, with a duty cycle of 100%.

Case	Total compliance (Nm/rad)	$\lambda_{\text{passive}}$ (%)	Control frequency (Hz)	Delay (ms)
1	1.6717	100	1,000	0
2	1.6717	100	1,000	0
3	1.6717	0	1,000	0
4	1.6717	0	1,000	17
5	2.5076	67	200	27
6	2.5076	67	100	27
7	2.8419	59	100	35

The case-1 robot simulated a compliance ratio of 1.0, i.e., fully passive elastic knee joints. The robot was dropped from a height of 0.7 m and landed successfully. The case-2 robot used identical control parameters, was dropped from 1.0 m height, and failed to land successfully. At close observation it becomes visible that its knee joints inverted after the first landing rebound, and the robot landed with inverted knee angles and without spring support. Case-2 emphasizes the drawback of passive compliance; without control, the knee joint orientation cannot be adjusted prior to rebounding. The case-3 configuration featured a controller with full, bi-directionally active compliance (no passive compliance), and without sensorimotor delay. The controller ran at 1 kHz and successfully guided the landing. In case-4, a fully active compliant robot with 17 ms sensorimotor delay failed to land properly, which shows the vulnerability of active compliance in the presence of sensorimotor delay. Case-5 shows a successful landing scenario by combining passive and active compliance ($\lambda_{\text{passive}}=0.67$), with 27 ms sensorimotor delay, and reduced control frequency (200 Hz). Case-6 was also configured with a $\lambda_{\text{passive}}=0.67$, a control frequency of 100 Hz, and failed landing the robot. For case-7, we decreased the compliance ratio to $\lambda_{\text{passive}}=0.59$, and the robot landed successfully from a height of 1.2 m, and with a sensorimotor delay of 35 ms at a control frequency of 100 Hz. Case-7 shows how an appropriate combination of active and passive compliance at low control frequency maintains good control authority and robustness in the presence of sensorimotor delay.

### 3.4 Hardware Experiments

We validated the previous single-leg simulations with hardware experiments. We chose compliance ratios of $\lambda_{\text{passive}}=\left[0\;,0.37\;,0.67\;,1\right]$ and a total rotational knee stiffness of $K_{\text{total}}=1.67\;\text{Nm}/\text{rad}$. We then varied control frequencies ([1000, 100, 10]Hz) and sensorimotor delays ([0, 10, 20, 30, 50]ms). The duty cycle was set to 50% for 10 and 100 Hz control frequency, and 100% for 1,000 Hz control frequency.

**FIGURE 9:**
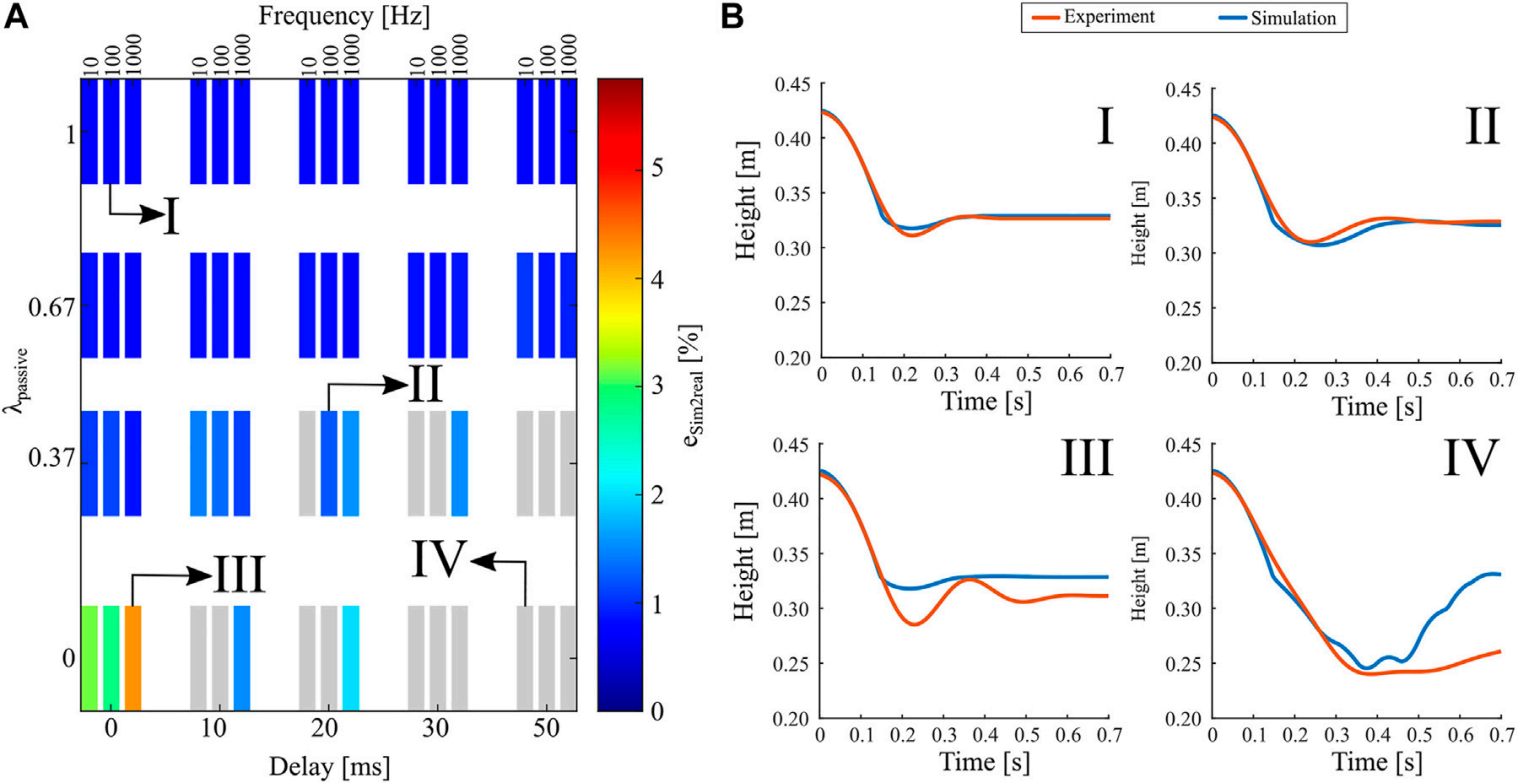
Results comparing computer simulations and hardware experiments, as root-mean-square error of the instantaneous hip height normalized by the initial leg length. **(A)** Good similarities are shown as colored data patches. Grey data patches indicate unsuccessful drop experiments, violating settling time or final height criteria. **(B)** Hip trajectories. (I–III) Successful landings with short settling times and sufficient settling hip heights. (IV) An example of an unsuccessful landing in simulation and hardware.

In Figure 9, we assess the difference $e_{\text{sim}2\text{real}}$ between computer simulations and hardware experiments, as the root-mean-square error (RMSE) between two resulting hip trajectories, normalized by the maximum leg length, measured during the settling duration of 0.7 s. The criteria for successful drop landings in hardware and computer simulation are identical (Section 3.2). Grey colored data shows failure cases in both experiments and simulations. Viable cases with an RMSE of less than 6% (Figure 9) indicate good consistency between hardware experiment and computer simulation. We show four exemplary hip trajectories for varying compliance ratios (Figure 9, I–IV). The first two cases are feasible landings with good consistency between simulation and experiments. In case III, the hardware experiment stabilized at a lower-than-simulation hip height but still within the required margin. Case IV is a failed drop, and neither the hardware experiment nor the simulated robot leg showed the necessary settling behavior.

## 4 Discussion

The single-leg drop results in Figure 5 show a continuous and gentle decrease of system robustness with increasing feedback delay when transitioning from a fully physically springy leg toward a fully actuated leg controlled by a virtual-spring controller. Hence, parallel structures of active compliance with the correct amount of passive compliance offer one possible answer to the question of how animals counteract perturbations in the presence of large sensorimotor delays. The ratio of passive to active compliance could be permanently set genetically, formed over a lifetime by training (Fouré et al., 2012), or set when required by partial or full recruitment of slacked muscle-tendon structures (Hogan, 1984). Legged robots equally benefit from intrinsic robustness against feedback delay. We believe that compliance ratios for other designs will depend on the specific leg and controller design parameters, the locomotion task, and the required controllability. If the available control frequency is limited or high delays are expected, a higher compliance ratio can be used. In the future, we are especially interested in exploring compliance rations of $\lambda_{\text{passive}}>0.5$. One early design choice to consider is the effective spring deflection. Typically, stiffer springs feature a smaller deflection range possibly leading to limited joint movement range, compared to softer springs. We used springs designed for large deflections. One can also balance the knee cam radius with the spring’s movement range and stiffness. We suggest the following, general procedure to establish a compliance ratio for a given task and robot:

1. Select a total joint stiffness based on the required steady state leg length, the maximum leg deflection, and the leg geometry (segment lengths, robot mass, cam radius). This step can be executed by test-mounting a λ=1 spring (no actuator) with given stiffness and spring slack position, dropping the robot leg, and observing its joint angles.
2. Alternatively, a simplified kinematic model can provide an estimate of the steady-state leg length (Supplementary Material Section 3).
3. Select a desired compliance ratio. In the examples shown, compliance ratios between 0.5 and 0.7 worked well. Low compliance ratios provide higher controllability, as long as control frequencies are high and feedback delays are low, and vice versa. Duty cycles should be set to maximum (100%), unless they are specifically exploited.
4. Check that the parallel mounted actuator has the capacity to supply the required torque and speed. Low compliance ratios (≈0.5) require an actuator providing a higher work and power output throughout the task. With higher compliance ratios (>0.7) the parallel spring carries more base load. When spring dynamics must be overwritten, high actuator torques are required but typically for shorter time. For a motor-gearbox design methodology we refer to Roos et al. (2006).

This work centers around adjusting the ratio of physical, passive compliance for a given total joint compliance. Online-adjustable spring stiffness mechanisms have been proposed, but many are still bulky and heavy (Yamaguchi and Takanishi, 1997; Vanderborght et al., 2013; Wolf et al., 2015). If a locomotion task requires large changes of total joint stiffness with a constant ratio of passive compliance (Ferris et al., 1998), robust and light-weight adjustable stiffness designs will be needed. For versatile locomotion sequences like jumping, landing and fast running, learning-based methods could extract a ‘best’ range of compliance ratios from large locomotion data sets.

We see at least three applications for hybrid compliance ratios in legged robots; 1) For legged robots which exploit natural dynamics of mechanical springs but require intermittent, high controllability for tasks like jumping or acceleration (Spröwitz et al., 2013; Lakatos et al., 2017). 2) For legged robots without access to high-frequency control or low-noise and low-latency sensors, which are expensive and time-consuming to develop (Nam et al., 2020). 3) For motion planners featuring update frequencies in the low sub-kilohertz range, in need of a legged robot with intrinsic robustness when controlled at these frequencies (Ponton et al., 2018).

## 5 Conclusion and Summary

We systematically characterized combinations of parallel mounted passive and active joint compliance for their ability to control the robot’s leg length after landing. We tested against detrimental effects of significant feedback delays, low control frequencies, and low duty cycles in the full range of compliance ratios. Our goal was to find a compliance ratio for one given total knee compliance that works well with the above controller limitations. In comparison, previous work in parallel-elastic legged robotics typically investigated parallel compliance with high-frequency and low delay actuation (Batts et al., 2016; Mazumdar et al., 2016).

Our computer simulations show successful single-leg drop-landings for sensorimotor delays up to 60 ms, and control frequencies as low as 20 Hz in combination with a compliance ratio of $\lambda_{\text{passive}}=0.7$. For a ‘hybrid’ setting between $\lambda_{\text{passive}}$ 0.4 and 0.7; the partially active compliance ensures good control authority, and the remaining passive, spring-based compliance reacts immediately and independently from the controller. We verified single-leg computer simulations with hardware experiments for a range of parameters and showed good agreement between both.

We ran computer simulations of quadruped robots with varying total leg stiffness values when landing from multiple drop heights. Compliance ratios in the hybrid range (around 0.5) worked better in the presence of adverse controller settings (delays, control frequency) than active compliance, and allowed for the necessary amount of controllability compared to pure passive compliance. We finally note that the engineered compliance ratios were robustly handling feedback delays similar to the neuromuscular sensorimotor delays reported of running animals of equal size to the presented hybrid robot leg.

## Data Availability

The raw data supporting the conclusion of this article will be made available by the authors, without undue reservation.
