# Supplementary material for: Hybrid Parallel Compliance Allows Robots to Operate With Sensorimotor Delays and Low Control Frequencies
Source: Front Robot AI. 2021 Jun 16;8:645748. doi: 10.3389/frobt.2021.645748 (PMC8302765; doi:10.3389/frobt.2021.645748)
Supplement: Supplementary file 1 [file DataSheet1.PDF]

Supplementary Material

1 PYBULLET SIMULATION, DETAILS CONTACT MODEL

The PyBullet simulator was used, and Bullet applies a hard contact model (Jean, 1999). For the ground plane, the model parameters are: lateral friction 10.0, restitution 0.0. The robot parameters: linear damping 0.04, angular damping 0.04, restitution 0.0, lateral friction 0.00022.

2 PYTHON CODE SNIPPET, MULTI-BODY SIMULATION

The code snippet for the implementation of delay and control frequency for hybrid active and passive compliance in knee joint, in Python:

```
import numpy as np
# creating an object of the simulated leg in the corresponding simulator that includes two DoF leg
robot = CreateObjectOfSingleLeg()
tau = np.array([0, 0]) #initialize the input torque vector

#the rest angle of spring
rest_angle=0.3288/2
#initialize Hip desired angle
q_des[0] = -1*restAngle/2
#initialize knee desired angle
q_des[1] =  1*restAngle
#initialize desired angular velocity
dq_des = np.array([0, 0])

#reset state of the simulated robot
robot.reset_state(q_des, dq_des)

#create and initialize the knee joint torque
motor_torque = np.zeros(1)

#create the buffer array for the  knee joint torque for applying the delay
motors_torque_buffer = np.empty((0, 1))

#create temporary torque for knee joint
motor_torque_temp = np.array([[0.0]])

#create an buffer array for the  applying time of knee joint torques
applying_timing_buffer=np.array([])

#create an array for the time index of applying time vector of knee joint torque
applying_timing_buffer_int=np.array([],int)

#total stiffness
k_stiffness=4680
# ratio of passive stiffness
lambda = Percentage
#sensorimotor delay
delay= Delay
#control loop frequency
frequency=Frequency #hz
```

```
time_simulation=0.001
radius_spring_pulley=0.0189 #meter
control_period=(1000/frequency)
#the number for control cycle of applying torque during a control period
torque_applying_number=(1000/frequency)*duty_cycle
#an index number for applying torque during a control period
index_torque_applying_number=1000


#simulation loop
for i in range(simulation_total_time):
    #condition for applying the torque corresponding to duty cycle
    if(index_torque_applying_number<torque_applying_number):
        index_torque_applying_number=index_torque_applying_number+1
    else:
        motor_torque=np.zeros(1)
        index_torque_applying_number=index_torque_applying_number+1

    #receiving the states of the leg from simulator
    q, dq = robot.get_state()
    active_eff, forces = robot.get_force()

    #implementation of control frequency and delay
    if (frequency!=0):
        if ((i%(1000/frequency))==0):
            applying_timing_buffer=np.append(applying_timing_buffer,time_simulation+delay*0.001)
            applying_timing_buffer_int=np.append(applying_timing_buffer_int,i+delay)

            motor_torque_temp[0][0] = (1.0-lambda)*k_stiffness*radius_spring_pulley*radius_spring_pulley*(q[2]- q_des[2])
            motors_torque_buffer= np.append(motors_torque_buffer,motor_torque_temp, axis=0)

    if (applying_timing_buffer_int.size != 0):
        if(applying_timing_buffer_int[0]==i):
            index_torque_applying_number=1
            applying_timing_buffer_int=np.delete(applying_timing_buffer_int,0)
            motor_torque=motors_torque_buffer[0,:]
            motors_torque_buffer=np.delete(motors_torque_buffer,0,0)

    #hip joint follows half of the knee joint angle as a desired value to constraint the leg motion
    q_des[0]=-1*q[1]/2
    #pd controller on the hip joint
    tau = -(3.0 * (q-q_des) + 0.07* dq)

    #simulating the spring torque
    spring_torque = np.zeros(1)
    spring_torque[0]= (1*lambda*k_stiffness*radius_spring_pulley*radius_spring_pulley*(q[2]-q_des[2]))
    if(q[2]<(rest_angle)):
        motor_torque[0]=0.0
        spring_torque[0]=0.0

    tau[1]= -1*spring_torque[0] - motor_torque[0]

```

```
        #sending the torque command to the robot
        robot.send_joint_command(tau)
        time_simulation=time_simulation+dt

```

Listing 1: Python code snippet for the simulation

3 JOINT TORQUE AT STEADY-STATE, DUTY CYCLE VARIATIONS

We estimate the steady state hip height of the two-segmented robot leg, based on a simplified model; all masses are concentrated in the hip joint and segments are massless. No friction is assumed. We also assume steady state conditions without dynamics or feedback. Instead, the torque acting on the knee joint, caused by the spring, and by the robot mass are calculated, according to (Biewener, 1991).

The intersections between dashed and solid lines in Figure S1 indicate the steady-state hip height for varying effective knee stiffness values (normalized, $K_{\text{sum}}/K_{\text{max}}$). The two-segmented leg configuration is nonlinear, especially for the shown, erect leg posture.

The $100\,\%$ (maximum) knee spring stiffness is $K_{\text{max}} = 4680\,\text{N/m}$, the knee cam radius is $r = 18.9\,\text{mm}$, the spring is slack at a knee angle of $\theta_{\text{slack}} = 170°$. The rotary stiffness K_{max} is then $1.67\,\text{Nm/rad}$. The knee spring and the active compliance (for a given duty cycle DC) exert torque into the knee joint, for knee angle changes of $\Delta\theta = \theta_{\text{slack}} - \theta$ in $[\text{rad}]$:

$$\tau_{\text{knee,extending}} = ((1 - \lambda_{\text{passive}})DC + \lambda_{\text{passive}}) * K_{\text{max}} * r^2 * \Delta\theta \tag{S1}$$

Hip height h_{hip} is a function of the knee angle θ, the segment lengths ($l = 0.16\,\text{m}$), and the foot radius ($r_{\text{foot}} = 15\,\text{mm}$).

$$h_{\text{hip}} = (2 * l * \sin(\theta/2)) + r_{\text{foot}} \tag{S2}$$

The robot mass m exerts a knee flexing torque, which depends on the knee angle, and the segment length:

$$\tau_{\text{knee,flexing}} = m * 9.81 m/s^2 * l * \cos(\theta/2) \tag{S3}$$

At steady-state the extensor and flexor torques are balanced, visible as intersections in Figure S1.

$$\tau_{\text{knee,extending}} = \tau_{\text{knee,flexing}} \tag{S4}$$

We show three steady-states in Figure S1:

1. Full hip torque applied (purple line, $K = 100\,\% K_{\text{max}}$),
2. A duty cycle $DC = 50\,\%$ and a $\lambda_{\text{passive}} = 0.60$, the sum of K amounts to $K = (0.60 + (0.50 * (1.0 - 0.60)))K_{\text{max}} = 0.8K_{\text{max}}$ ($80\,\%$, yellow line)
3. A duty cycle $DC = 25\,\%$ and a $\lambda_{\text{passive}} = 0.45$, the sum of K amounts to $K = (0.45 + (0.25 * (1.0 - 0.45)))K_{\text{max}} = 0.59K_{\text{max}}$ ($59\,\%$, red line).

All three intersections are above the steady-state hip height threshold of $0.30\,\text{m}$. Figure S2 shows that a net stiffness of $K = 34\,\%$ leads to a steady-state hip height of $0.30\,\text{m}$.

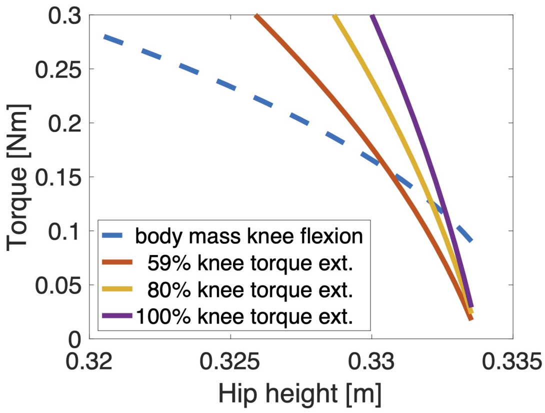

Figure S1: The blue, broken line shows the flexing torque exerted by the body weight onto the knee joint, for a range of hip heights. Full lines show different knee extensor configurations, based on a physical or virtual spring with slack angle $170°$ (hip height $334\,\mathrm{mm}$). The intersections with the broken line indicate the steady-state hip height for three indicated knee actuator configurations.

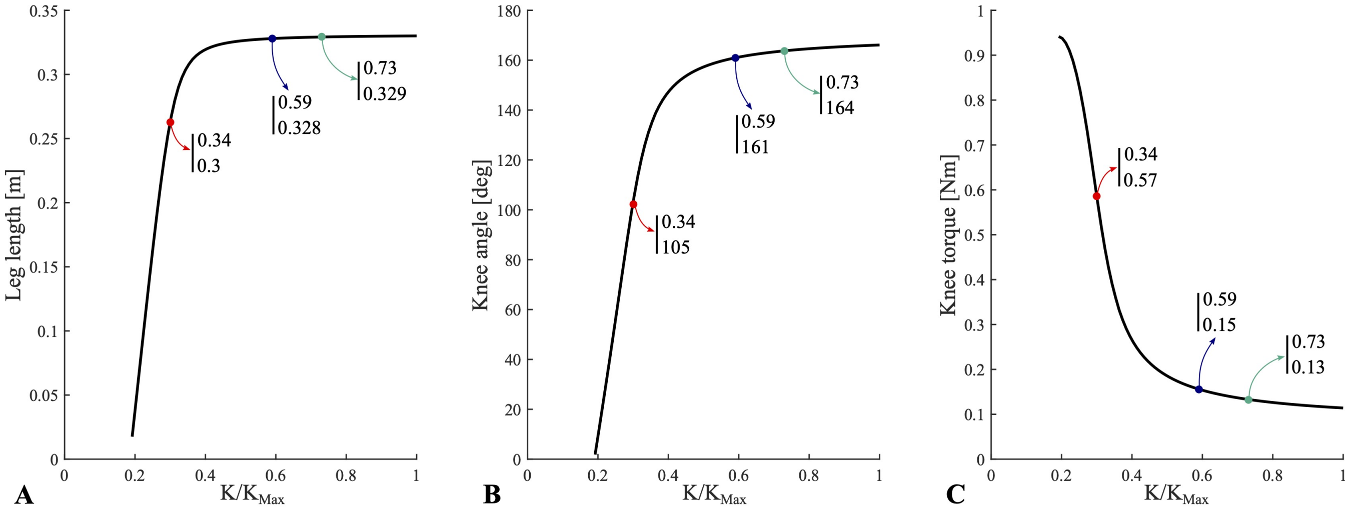

Figure S2: A) The steady state leg length (hip height) for varying levels of $K_{\mathrm{sum}}/K_{\mathrm{max}}$. (B) The same but for knee angle, and (C) for knee torque. The upper value indicated by the data marker pair is the exact x-value, the lower the y-value. All three solutions indicated lead to steady-states of at least $0.3\,\mathrm{m}$ hip height.

4 DISCRETE-TIME ANALYSIS

This section shows results of a discrete-time pole analysis and step response of a reduced-order model, for a simplified pendulum example. We investigate the effect of varying sample times (control frequencies), $\lambda_{\mathrm{passive}}$ values, and feedback delays. We apply the *c2d* Matlab function for discretization with zero order hold method (*ZOH*), plot data on the z-plane and calculate the system step response in the discrete mode. We apply the Matlab discretization toolbox to map the system's transfer function from the s-plane to the z-plane. In the z-plane, the system is stable when poles are inside the unit circle around the origin. By increasing $\lambda_{\mathrm{passive}}$ the system becomes more stable (Figure S3). The pendulum settles faster for higher $\lambda_{\mathrm{passive}}$ (Figure S4). As expected, increased delay destabilizes the simplified pendulum model, for low $\lambda_{\mathrm{passive}}$.

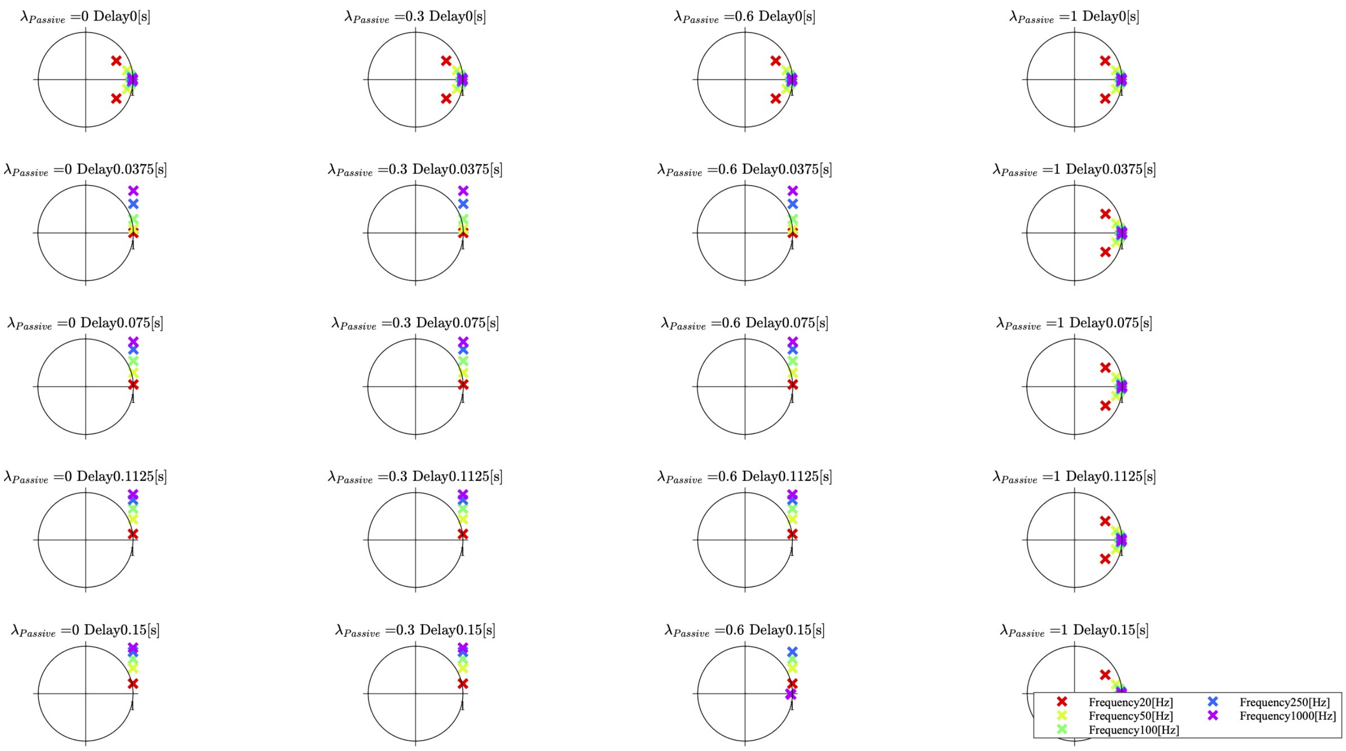

Figure S3: Discrete-time pole analysis for frequencies $[20,\ 50,\ 100,\ 250,\ 1000]\mathrm{Hz}$, $\lambda_{\mathrm{passive}}$ $[0.0,\ 0.3,\ 0.6,\ 1.0]$, and delay $[0,\ 37,\ 75,\ 112,\ 150]\mathrm{ms}$. Poles inside the unit circle indicate stable pendulum movements, otherwise the feedback delay destabilizes the pendulum movement.

5 DERIVATE TERM

For the interested reader, we added a derivative term (marked as bold) to the active compliance controller (Equation 6), leading to:

$$\tau_{\mathrm{knee,motor}} = (1-\lambda_{\mathrm{passive}})(K_{\mathrm{total}}(\theta_{\mathrm{feedback,knee}}-\theta_{0,\mathrm{knee}}) + \mathbf{D}(\dot{\theta}_{\mathrm{feedback,knee}})) \tag{S5}$$

We simulated D gains of 0.02 (Figure S5A) and 0.06 (Figure S5B). We implemented $\lambda_{\mathrm{passive}}$ from 0.0 to 1.0 in steps of 0.05, sensorimotor delay from $0\,\mathrm{ms}$ to $60\,\mathrm{ms}$ in steps of $5\,\mathrm{ms}$, and sensorimotor control frequencies of 20, 50, 100, 250, and $1000\,\mathrm{Hz}$.

In combination with the virtual spring model controller, the D-term somewhat acts like that of a proportional-derivative controller. With a D gain of 0.02 feasible regions for all control frequencies increase, as expected from the damping-style properties and the drop landing task requirements. For higher D gain (0.6) the feasible region increases for high control frequencies. At low control frequencies ($20\,\mathrm{Hz}$ and $50\,\mathrm{Hz}$) and low sensorimotor delays, low compliance ratio regions become unfeasible; here the system starts oscillating.

We however note that Figure S5 is not really presenting a fair comparison, compared to non-D value compliance ratios; the active compliance plus derivate is over-pronounced compared to the mechanical, physical compliance, and its speed dependency invalids a side-by-side comparison of drop landings. Time-derivative terms are brittle in the presence of noise, hence sensor noise should be simulated for these terms.

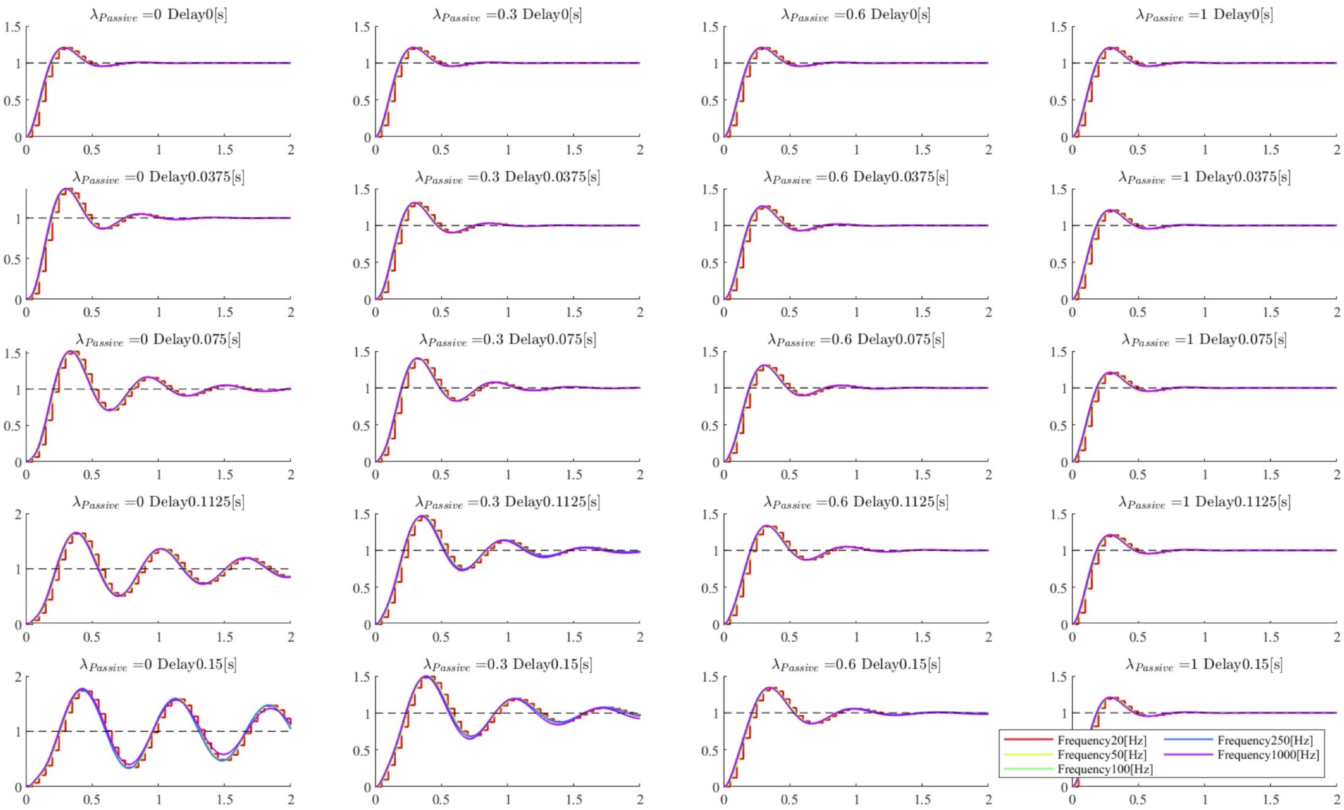

Figure S4: Discrete-time modeling of a simplified pendulum movement for frequencies $[20,\ 50,\ 100,\ 250,\ 1000]\text{Hz}$, λ_{passive} $[0.0,\ 0.3,\ 0.6,\ 1.0]$, and delay $[0,\ 37,\ 75,\ 112,\ 150]\text{ms}$. Pendulum angles are shown over time.

REFERENCES

Biewener, A. A. (1991). Musculoskeletal design in relation to body size. *Journal of Biomechanics* 24, Supplement 1, 19–29. doi:10.1016/0021-9290(91)90374-V

Jean, M. (1999). The non-smooth contact dynamics method. *Computer methods in applied mechanics and engineering* 177, 235–257. Publisher: Elsevier

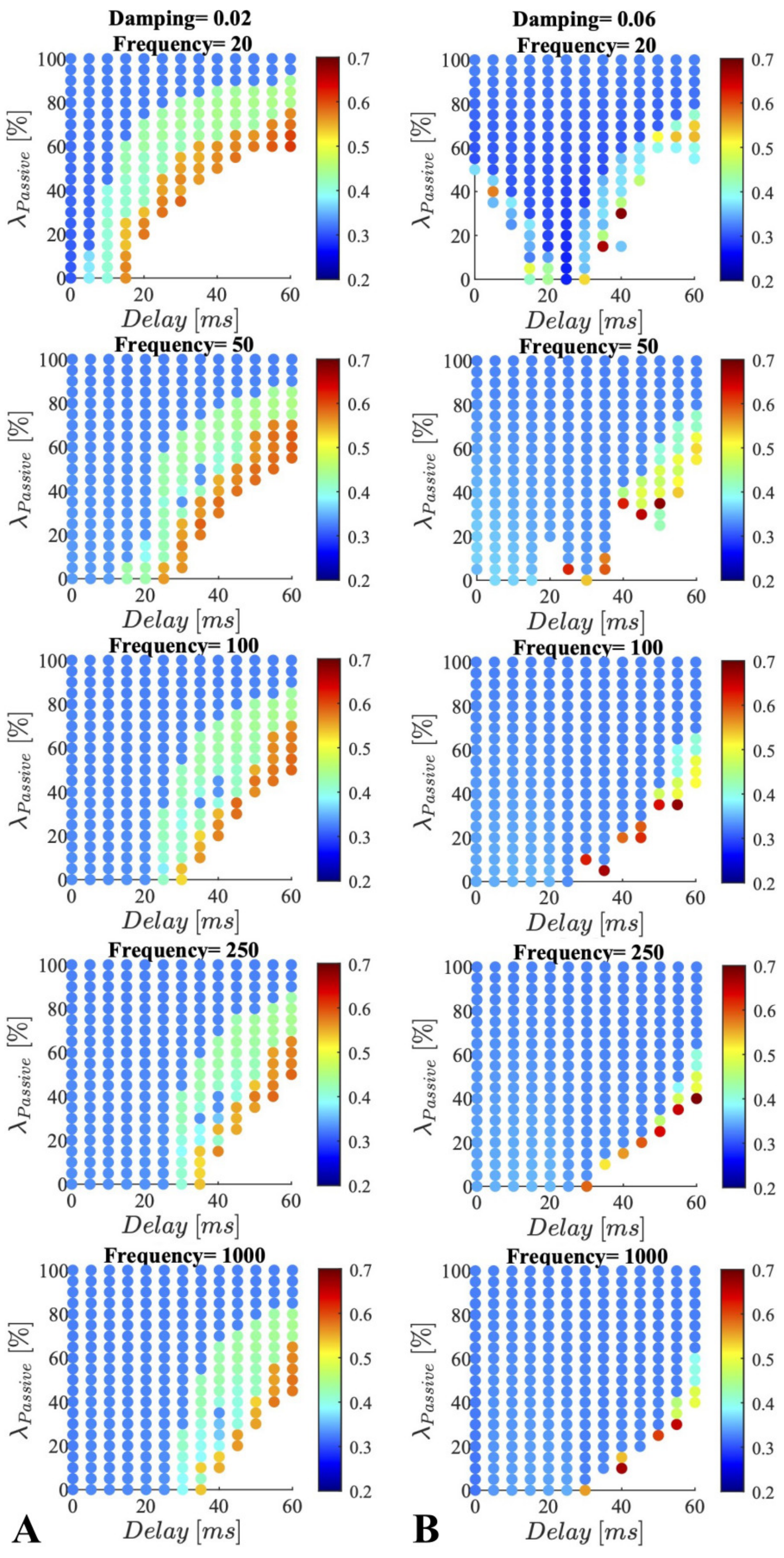

Figure S5: Results from adding a time derivative term to Equation 6 (active compliance), leading to Equation (S5). A) $D = 0.02$, and B) $D = 0.06$.
